# Supplementary material for: Transcriptional response to stress is pre-wired by promoter and enhancer architecture
Source: Nat Commun. 2017 Aug 15;8:255. doi: 10.1038/s41467-017-00151-0 (PMC5557961; doi:10.1038/s41467-017-00151-0)
Supplement: Supplementary file 1 — Supplementary Information [file 41467_2017_151_MOESM1_ESM.pdf]

File Name: Supplementary Information
Description: Supplementary Figures, Supplementary Methods and Supplementary References

File Name: Supplementary Data 1
Description: Nascent RNA-synthesis at genes. chr: chromosome, DownHC: down-regulated with high confidence (counted as down-regulated in the manuscript), DownLC: down-regulated with low confidence, DownNoTSS: down-regulated but no dREG-called regulatory element, dRPK: change in the RPK (HS-NHS), gb: gene body, geneName: the name of the gene, FC: fold change (HS/NHS), HS: 30 min heat shock at 42°C, NHS: non-heat shock conditions, Regulation: DESeq2-called regulation of the gene upon stress, pp: promoter-proximal, RefGeneName: transcript specification, RPK: reads per kilobase genomic DNA, strand: the strand of the coding sequence, TSS: transcription start site, TTS: transcript termination / polyA cleavage site, txEnd: transcript end site (RefGen), txStart: transcript start (RefGen), UnExp: unexpressed, UnReg: unchanged, UpHC: up-regulated with high confidence (counted as up-regulated in the manuscript), UpLC: up-regulated with low confidence, UpNoTSS: up-regulated but no dREG-called regulatory element.

File Name: Supplementary Data 2
Description: Distal Transcription Regulatory Elements (dTREs) as identified with dREG-HD. chr: chromosome, start / end: the coordinates of the dTRE, dTRE_group: condition(s) of dTRE-identification.

File Name: Supplementary Data 3
Description: HSF1-target sites grouped into distal Transcription Regulatory Elements (dTREs), active promoters, and genomic regions where no dREG-HD-identified TRE was detected. chr: chromosome, start / end: the coordinates of the HSF1 peak, HDgroup: genomic region of HSF1 target site, dTRE: dREG-HD called dTRE, HDprom: dREG-HD-called active promoter, noHD: HSF1 target site that do not occur on dTRE or HDprom.

File Name: Peer Review File
Description:

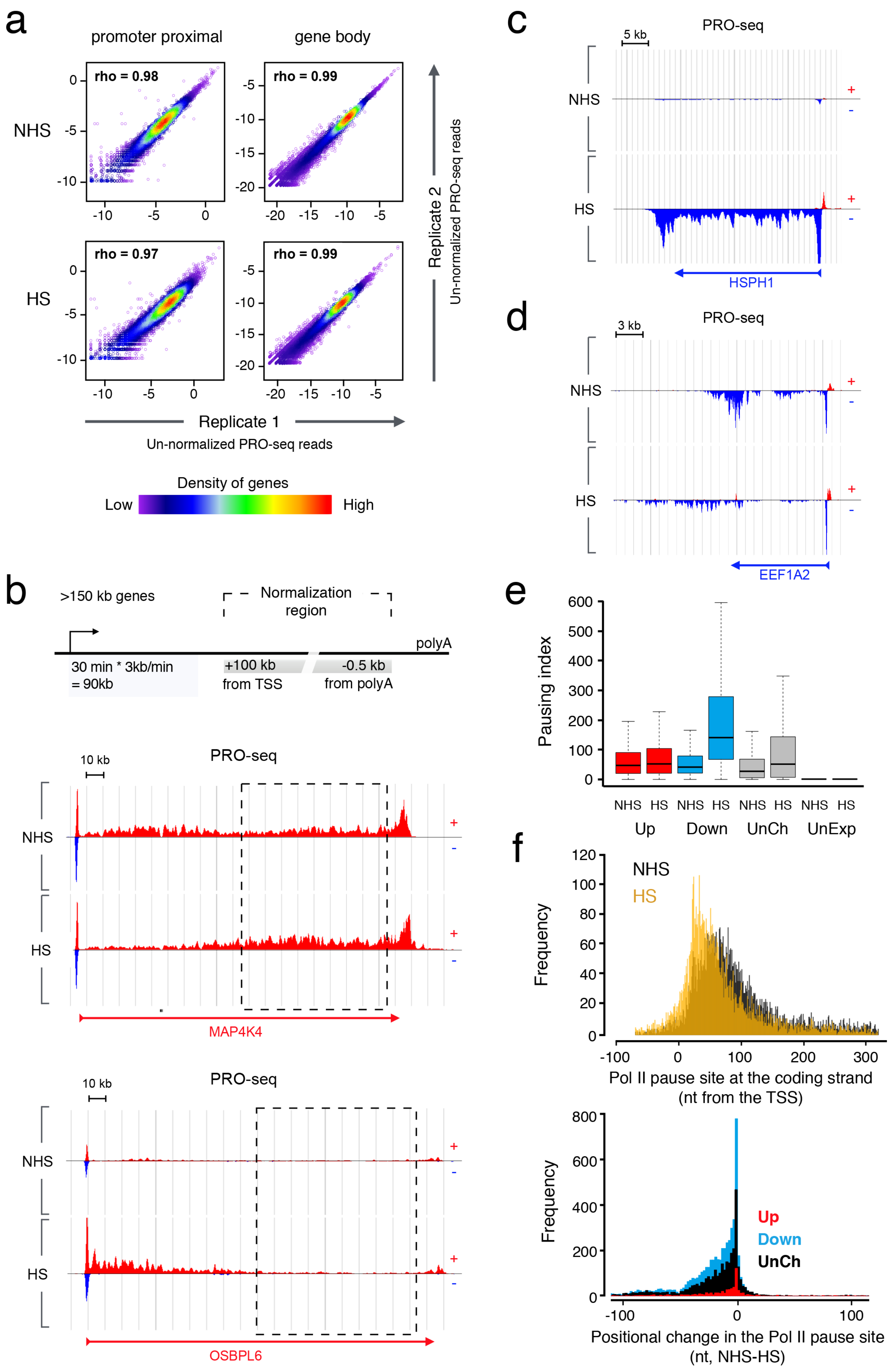
a
promoter proximal
gene body
NHS
HS
rho = 0.98
rho = 0.99
rho = 0.97
rho = 0.99
Replicate 2
Un-normalized PRO-seq reads
Replicate 1
Un-normalized PRO-seq reads
Density of genes
Low
High
b
>150 kb genes
Normalization region
polyA
30 min * 3kb/min = 90kb
+100 kb from TSS
-0.5 kb from polyA
PRO-seq
10 kb
MAP4K4
OSBPL6
c
PRO-seq
5 kb
HSPH1
d
PRO-seq
3 kb
EEF1A2
e
Pausing index
NHS
HS
Up
Down
UnCh
UnExp
f
Frequency
NHS
HS
Pol II pause site at the coding strand (nt from the TSS)
Up
Down
UnCh
Positional change in the Pol II pause site (nt, NHS-HS)

Supplementary Figure 1. Precision run-on sequencing (PRO-seq) reveals mechanisms of genome-wide reprogramming of transcription. a) Correlation of raw read counts from two biological PRO-seq replicates at promoter-proximal regions (left panels) and gene bodies (right panels) of the human genome for NHS (upper panels) and HS (lower panels) conditions. **b)** Biological replicates were normalized utilizing the 3' coding region (+100 kb from TSS to -0.5 kb from polyA site) of long (>150 kb) genes where the advancing or receding wave of transcription had not proceeded during 30 min of heat stress. The uppermost panel is a schematic presentation of the normalization, and the middle and the lowest panels illustrate long up-regulated and down-regulated genes, respectively, where the transcriptional change was not detected beyond 100 kb from the TSS. **c-d)** Genome browser images of transcriptionally engaged Pol II at **(c)** up-regulated *HSPH1*/*HSP110* and **(d)** down-regulated *EEF1A2* genes prior to (NHS) and upon (HS) acute heat stress. Transcription of the plus strand is indicated in red, minus strand in blue. **e)** Average pausing index at up-regulated, down-regulated and unchanged genes prior to (NHS) and upon (HS) heat shock. **f)** Upper panel: Distribution of Pol II pause sites prior to and upon acute stress for all genes with a detectable pausing. The lower panel displays the change in the Pol II pause site upon acute stress for up-regulated (Up), down-regulated (Down) and unchanged (UnCh) genes.

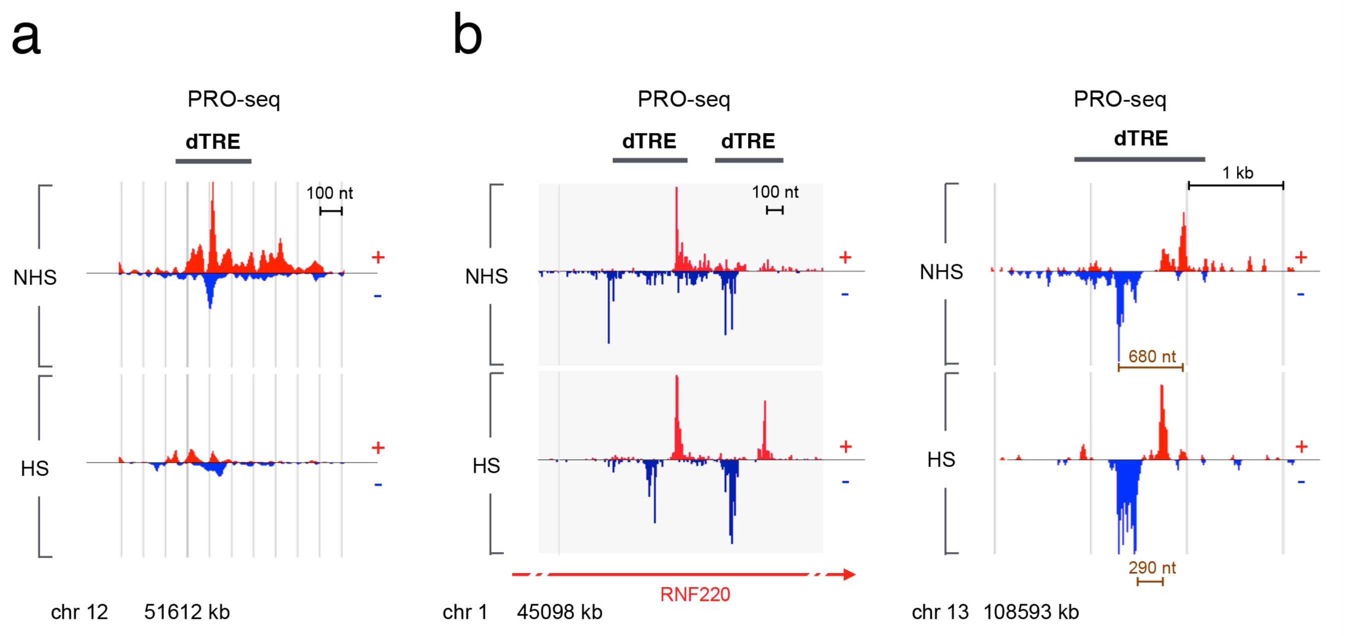

Supplementary Figure 2. The profile of engaged Pol II at dTREs changes upon stress. a-b) Browser shot images of dTREs with **a)** significantly reduced transcription or **b)** where the alignment pattern, but not the absolute read count, remarkably changed upon stress. Black bar above the browser shot represents the coordinates of the identified dTREs. The brown bars in the right panel depict the distance between the pause coordinates in NHS and HS conditions.

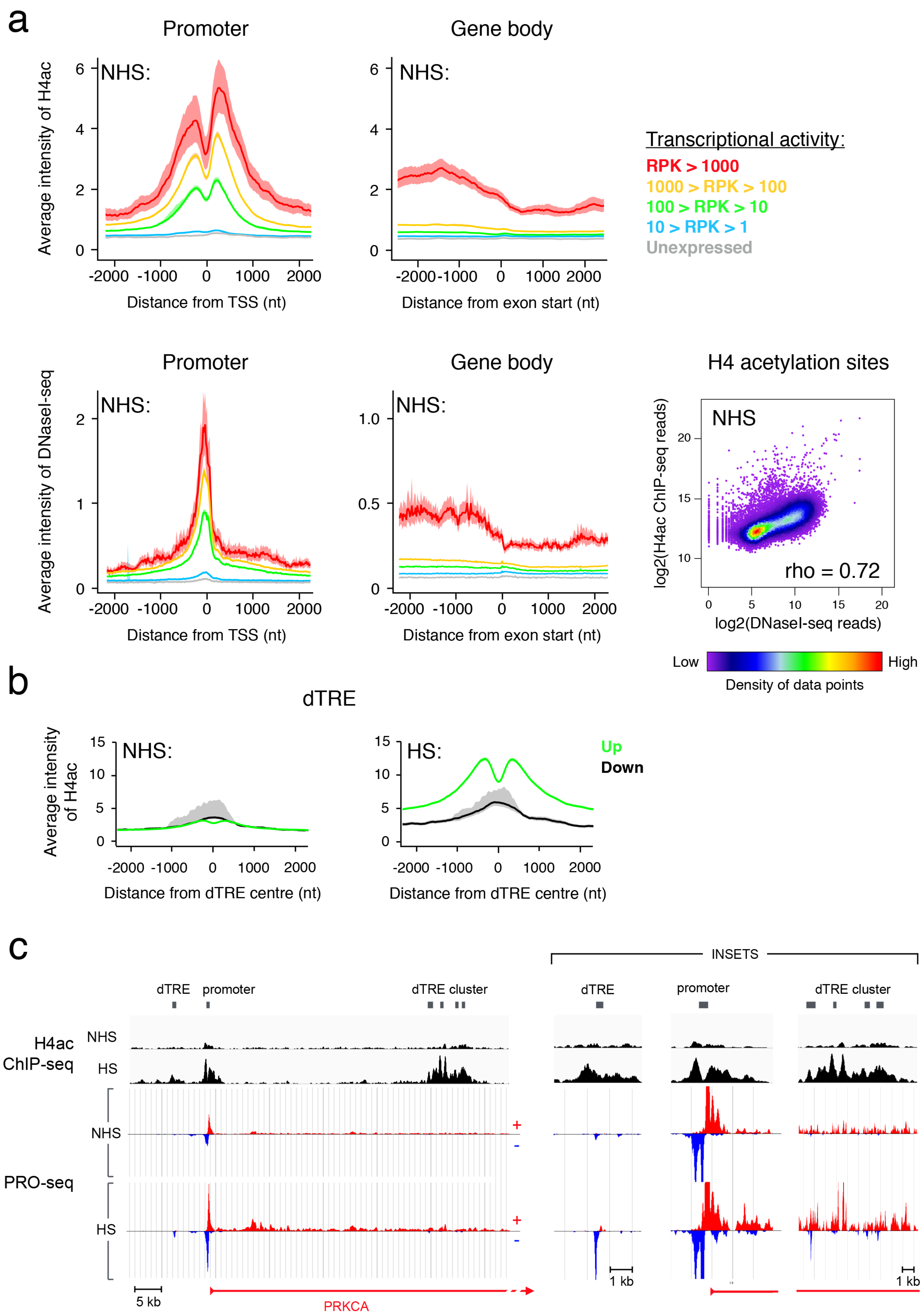

a
Promoter
Gene body
NHS:
Average intensity of H4ac
Distance from TSS (nt)
Distance from exon start (nt)
Transcriptional activity:
RPK > 1000
1000 > RPK > 100
100 > RPK > 10
10 > RPK > 1
Unexpressed
Average intensity of DNaseI-seq
H4 acetylation sites
NHS
rho = 0.72
log2(H4ac ChIP-seq reads)
log2(DNaseI-seq reads)
Low
High
Density of data points
b
dTRE
HS:
Up
Down
Average intensity of H4ac
Distance from dTRE centre (nt)
c
INSETS
dTRE
promoter
dTRE cluster
H4ac ChIP-seq
NHS
HS
PRO-seq
5 kb
1 kb
PRKCA

Supplementary Figure 3. Global change in the histone H4 acetylation state upon acute heat stress. **a)** Average intensity of histone H4 acetylation at promoters (upper left panel) and gene bodies (upper right panel) grouped by the genes' transcriptional activity. The profile of DNaseI hypersensivity at promoters (lower left panel) and gene bodies (lower middle panel) are shown as comparison, and the signal intensities of histone H4 acetylation and DNaseI hypersensitivity compared in a 2000 nt region centered to each histone H4 acetylation site across the genome (lower right panel). **b)** Average histone H4 acetylation at up- (green) and down- (black) regulated dTREs prior to (left panel) and upon (right panel) acute stress. **c)** Browser shot image of histone H4 acetylation (black) at 5' part of *PRKCA* gene, including an upstream dTRE, promoter and a dTRE cluster 40-50 kb downstream of the TSS. Transcriptionally engaged Pol II is indicated in red (plus strand) and blue (minus strand). DNaseI hypersensitivity data (DNase Digital Footprinting) is from the ENCODE[1].

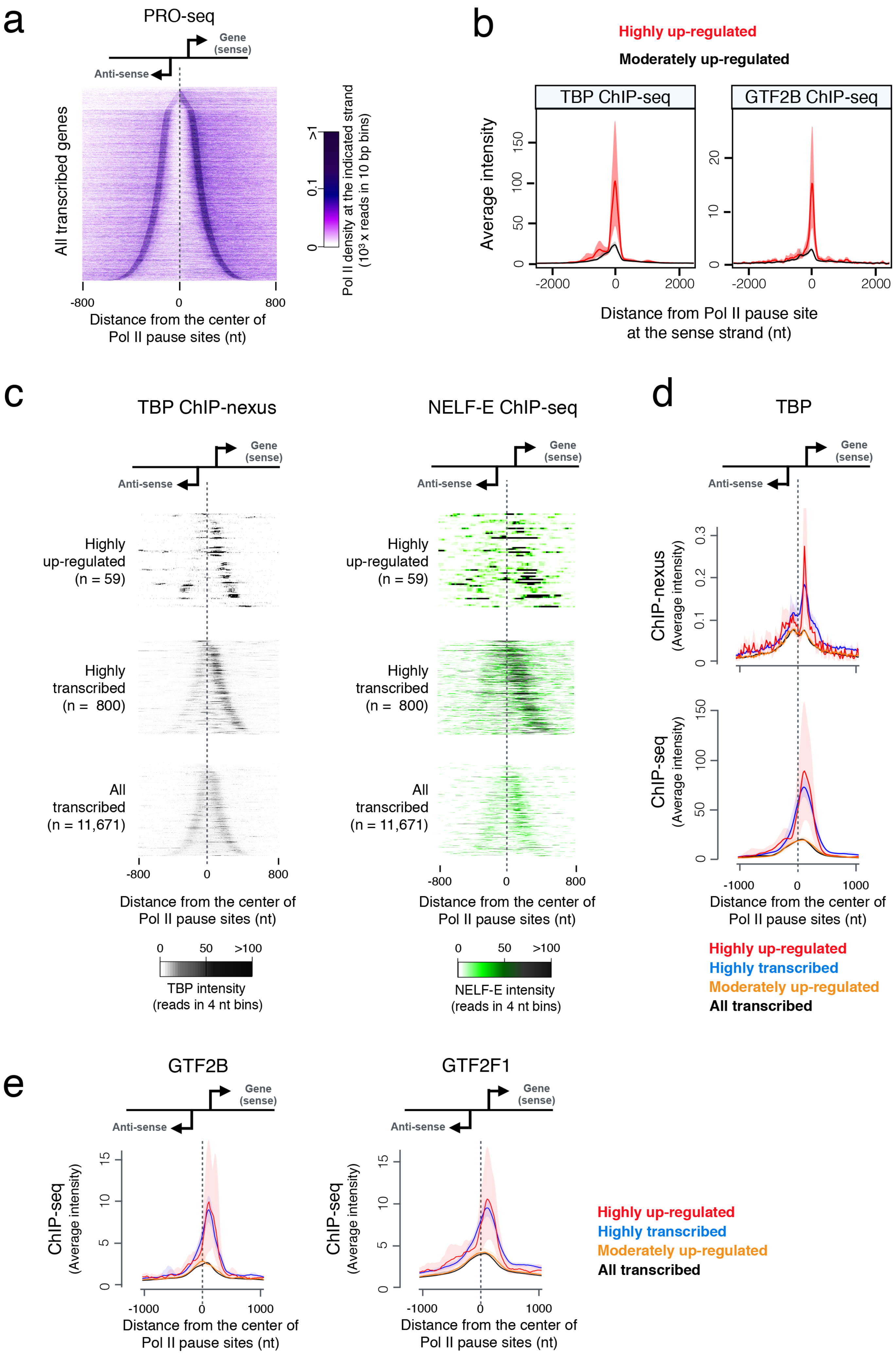
a
PRO-seq
Gene (sense)
Anti-sense
All transcribed genes
-800
0
800
Distance from the center of Pol II pause sites (nt)
0
0.1
>1
Pol II density at the indicated strand (10^3 x reads in 10 bp bins)
b
Highly up-regulated
Moderately up-regulated
TBP ChIP-seq
GTF2B ChIP-seq
Average intensity
-2000
0
2000
Distance from Pol II pause site at the sense strand (nt)
c
TBP ChIP-nexus
NELF-E ChIP-seq
Highly up-regulated (n = 59)
Highly transcribed (n = 800)
All transcribed (n = 11,671)
Distance from the center of Pol II pause sites (nt)
TBP intensity (reads in 4 nt bins)
NELF-E intensity (reads in 4 nt bins)
0
50
>100
d
TBP
ChIP-nexus (Average intensity)
ChIP-seq (Average intensity)
-1000
0
1000
Highly up-regulated
Highly transcribed
Moderately up-regulated
All transcribed
e
GTF2B
GTF2F1
ChIP-seq (Average intensity)
Distance from the center of Pol II pause sites (nt)
Highly up-regulated
Highly transcribed
Moderately up-regulated
All transcribed

Supplementary Figure 4. Chromatin architecture primes genes for rapid activation. a) Heat map of PRO-seq at all transcribed genes, centered on the mid point between the Pol II pause sites at the sense and the anti-sense strands. **b)** Comparison of TBP and GTF2B ChIP-seq intensities at the highly and moderately up-regulated genes. **c)** Heat maps of TBP ChIP-nexus and NELF-E ChIP-seq at highly up-regulated, highly transcribed and all transcribed genes. In heat maps, the genes are sorted by the increasing distance between the Pol II pause sites at the sense and the anti-sense strands, and the signal centered to the middle coordinate between the pause sites. The corresponding average intensities are shown in Fig. 4b,c. **d)** Comparison of signal intensities from ChIP-nexus and ChIP-seq for TBP. **e)** Average ChIP-seq signal intensities of GTF2B and GTF2F1 at highly up-regulated, highly transcribed, moderately up-regulated and all transcribed genes. The ChIP-seq datasets for TBP, GTF2B, GTF2F1 and NELF-E were obtained from the ENCODE[1], TBP ChIP-nexus is from He *et al*. (2015)[2].

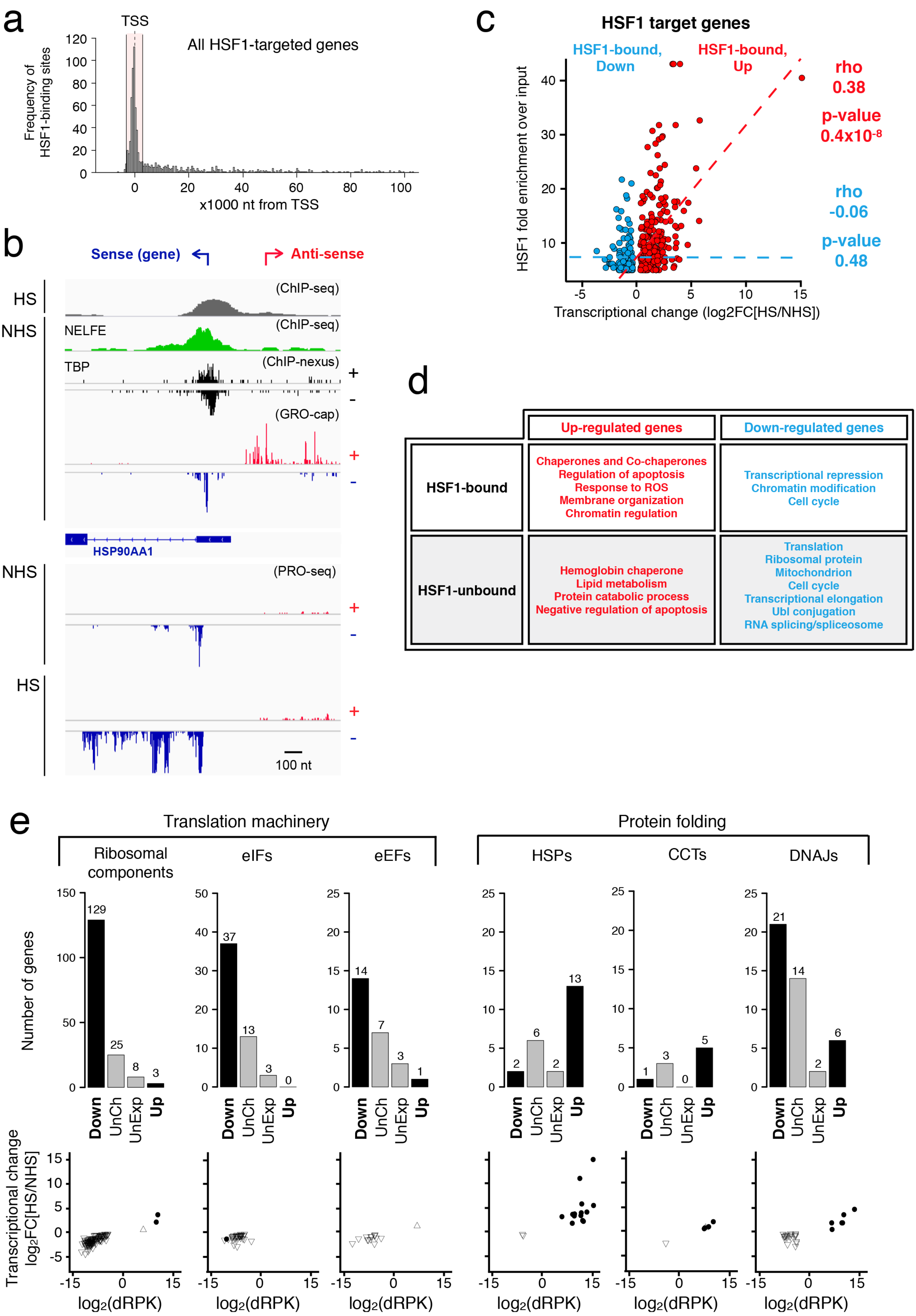
a
TSS
All HSF1-targeted genes
Frequency of HSF1-binding sites
x1000 nt from TSS
b
Sense (gene)
Anti-sense
HS
NHS
(ChIP-seq)
NELFE
(ChIP-seq)
TBP
(ChIP-nexus)
(GRO-cap)
HSP90AA1
NHS
(PRO-seq)
HS
100 nt
c
HSF1 target genes
HSF1-bound, Down
HSF1-bound, Up
rho 0.38
p-value 0.4x10-8
rho -0.06
p-value 0.48
HSF1 fold enrichment over input
Transcriptional change (log2FC[HS/NHS])
d
Up-regulated genes
Down-regulated genes
HSF1-bound
Chaperones and Co-chaperones
Regulation of apoptosis
Response to ROS
Membrane organization
Chromatin regulation
Transcriptional repression
Chromatin modification
Cell cycle
HSF1-unbound
Hemoglobin chaperone
Lipid metabolism
Protein catabolic process
Negative regulation of apoptosis
Translation
Ribosomal protein
Mitochondrion
Cell cycle
Transcriptional elongation
Ubl conjugation
RNA splicing/spliceosome
e
Translation machinery
Ribosomal components
eIFs
eEFs
Protein folding
HSPs
CCTs
DNAJs
Number of genes
Down
UnCh
UnExp
Up
Transcriptional change log2FC[HS/NHS]
log2(dRPK)

Supplementary Figure 5. HSF1 provokes transcriptional induction of chaperone machinery. a) Distribution of HSF1 binding sites at target genes. The histogram shows the frequency of HSF1-binding in each 500-nt window from the TSS. The colored area indicates +/-3000 nt from the TSS. **b)** Browser shot of HSP90AA1 gene indicating the positioning of heat-induced binding of HSF1, and the localization of NELF-E and TBP prior to stress, depicted with respect to the GRO-cap-identified transcription start sites at the sense and the anti-sense strands. The PRO-seq profile of transcriptionally engaged Pol II prior to (NHS) and upon (HS) heat stress is indicated below the gene. **c)** HSF1-binding intensity (fold enrichment over input) as the function of the gene's transcriptional response. The coefficients (rho) and p-values are according Spearman's rank correlation. The correlation lines are fitted for linear regression. **d)** Gene ontology analyses indicating enriched gene categories among up-regulated (red) and down-regulated (blue), HSF1-bound and HSF1-unbound, genes. **e)** The number of genes (upper panels) that encode components of the translation machinery or chaperoning proteins, annotated by the functional gene ontology and transcriptional response to stress. The lower panels depict the transcriptional change upon heat shock for each individual up- or down-regulated gene. HSF1-bound genes are indicated with closed circles and HSF1-unbound genes with open triangles. The ChIP-seq dataset for NELF-E was obtained from the ENCODE[1], TBP ChIP-nexus is from He *et al.* (2015)[2] and GRO-cap from Core *et al.* (2014)[3].

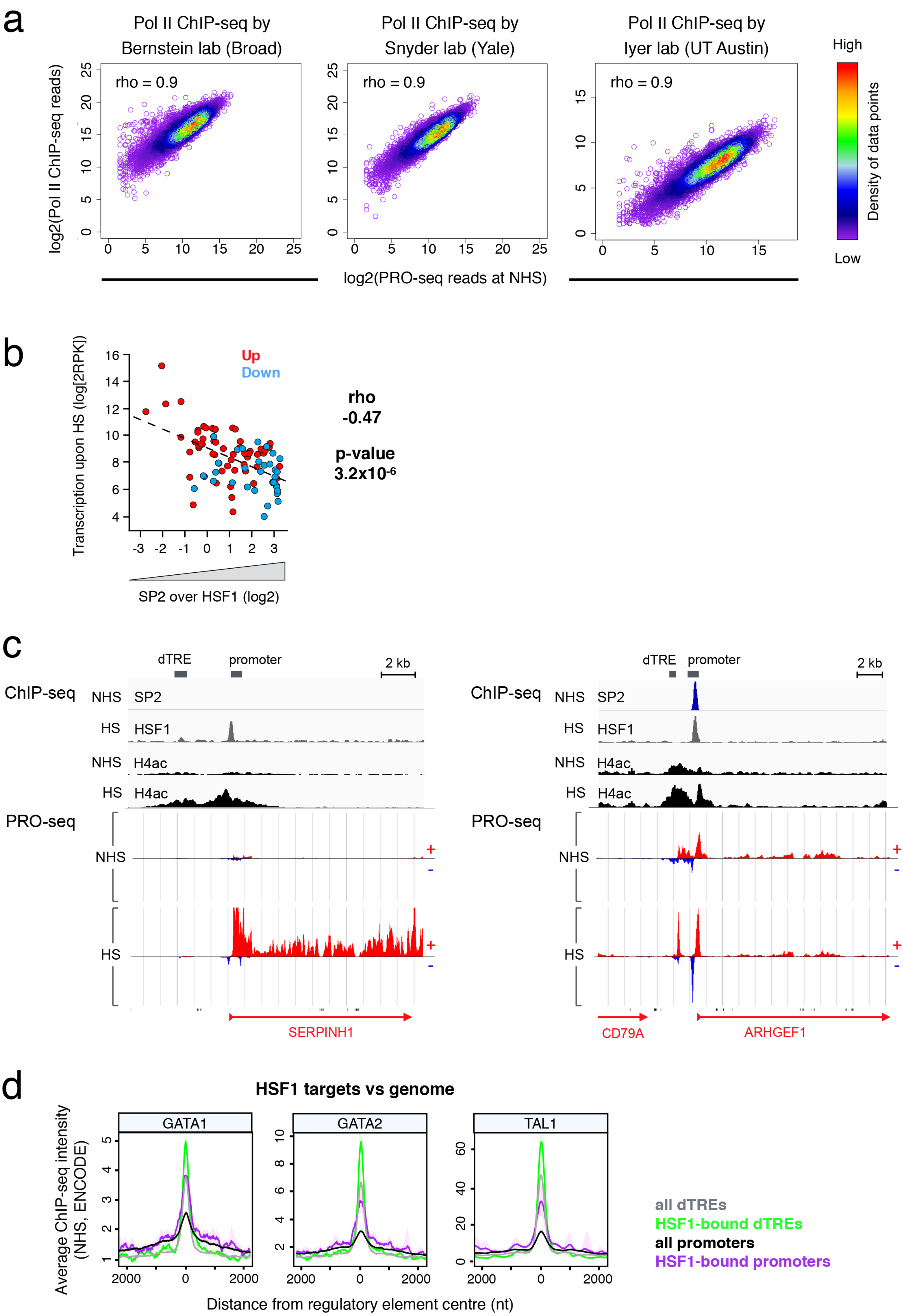

a
Pol II ChIP-seq by Bernstein lab (Broad)
Pol II ChIP-seq by Snyder lab (Yale)
Pol II ChIP-seq by Iyer lab (UT Austin)
rho = 0.9
log2(Pol II ChIP-seq reads)
log2(PRO-seq reads at NHS)
High
Density of data points
Low
b
Up
Down
rho -0.47
p-value 3.2x10-6
Transcription upon HS (log[2RPK])
SP2 over HSF1 (log2)
c
dTRE
promoter
2 kb
ChIP-seq
NHS SP2
HS HSF1
NHS H4ac
HS H4ac
PRO-seq
NHS
HS
SERPINH1
CD79A
ARHGEF1
d
HSF1 targets vs genome
GATA1
GATA2
TAL1
Average ChIP-seq intensity (NHS, ENCODE)
Distance from regulatory element centre (nt)
all dTREs
HSF1-bound dTREs
all promoters
HSF1-bound promoters

Supplementary Figure 6. Chromatin architecture can restrict HSF1-mediated *trans*-activation. a) Comparison of transcriptional profiles in non-stressed K562 cells using PRO-seq data of this study (x-axis), and Pol II ChIP-seq from Bernstein, Snyder and Iyer laboratories (y-axis of respective panels). The coefficient (rho) is according to Spearman's rank correlation. **b)** The ratio of SP2 over HSF1 plotted against the gene's transcriptional activity upon acute heat stress. The coefficient (rho) and p-value are according Spearman's rank correlation. The correlation line is fitted for linear regression. The statistics are calculated for all the genes plotted in the graph, the colors indicate the transcriptional response of the gene upon stress. **c)** Browser shot images of up-regulated gene of *SERPINH1* and down-regulated gene encoding *ARHGEF1*. **d)** Average intensities of GATA1, GATA2 and TAL1 at HSF1-targeted and all genomic dTREs and promoters. The ChIP-seq datasets for Pol II, SP2, GATA1, GATA2 and TAL1 were obtained from the ENCODE[1].

a

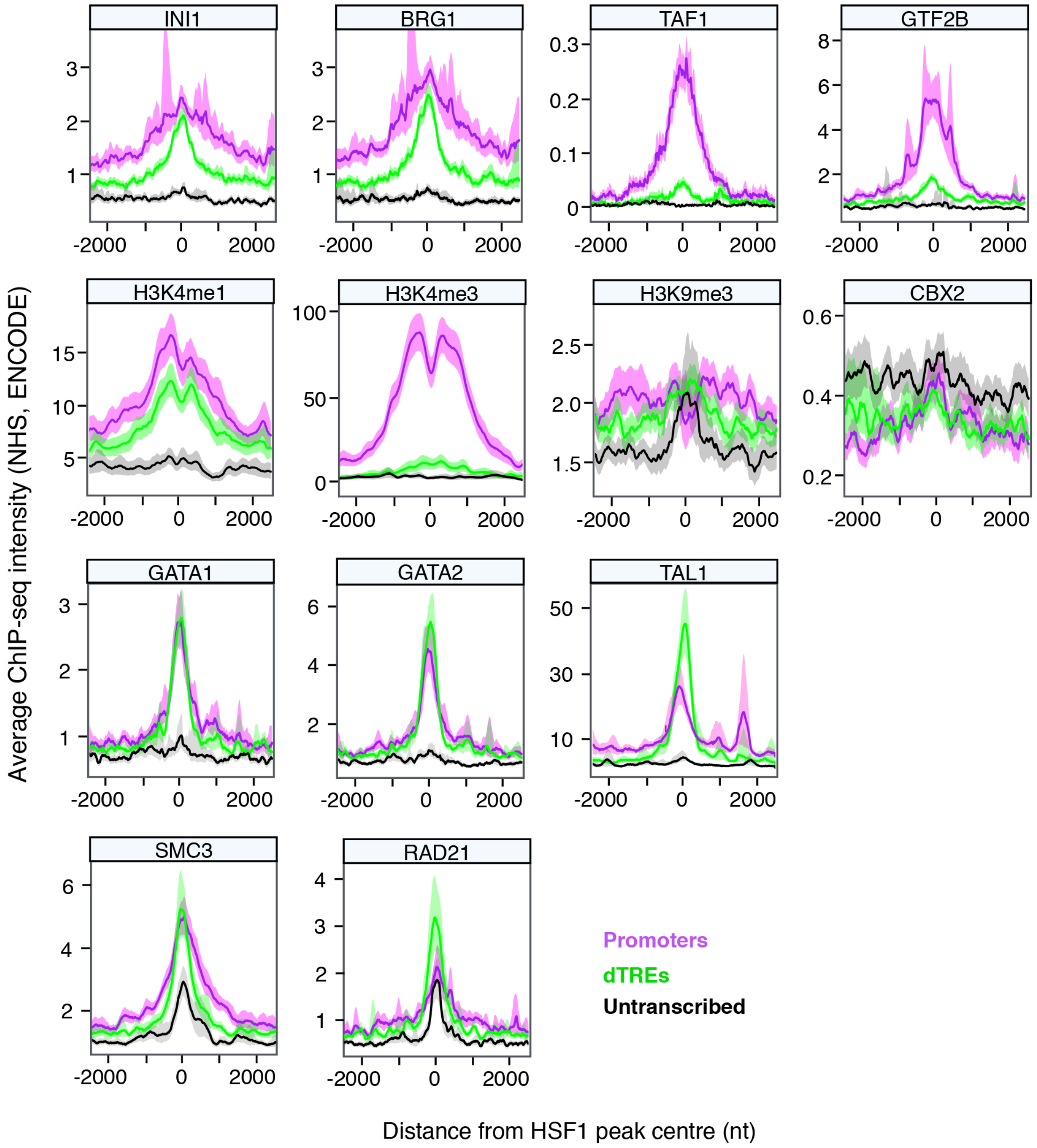

b

HSF1 targets sites

		Promoter	dTRE	Untranscribed
Structural proteins	CTCF	125	104	107
	SMC3	203	123	90
	RAD21	16	43	38
	ZNF143	316	225	109
Ubiquitous regulators of transcription	TBP	300	82	9
	TAF1	299	63	7
	GTF2B	206	43	5
	GTF2F1	115	15	8
	TAF7	68	5	8
	CCNT2	317	134	9
	NELFE	26	0	2
	SP1	136	46	6
	SP2	74	6	6
Cell type specific transcription factors	TAL1	253	225	48
	GATA1	25	30	5
	GATA2	55	92	8

Supplementary Figure 7. Distinct chromatin composition at HSF1-targeted promoters, dTREs and untranscribed regions. **a)** Average ChIP-seq intensities of chromatin remodelers (INI1 and BRG1), general transcription factors (TAF1 and GTF2B), histone modifications (H3K4me1, H3K4me3 and H3K9me3), a silencer (CBX2), cell-type specific transcription factors (GATA1, GATA2 and TAL1), and components of the cohesin complex (SMC3 and RAD21) at HSF1 target sites. **b)** The number of HSF1-targeted promoters, dTREs and untranscribed genomic regions that are occupied by the indicated protein in optimal growth conditions. All datasets were obtained from the ENCODE[1].

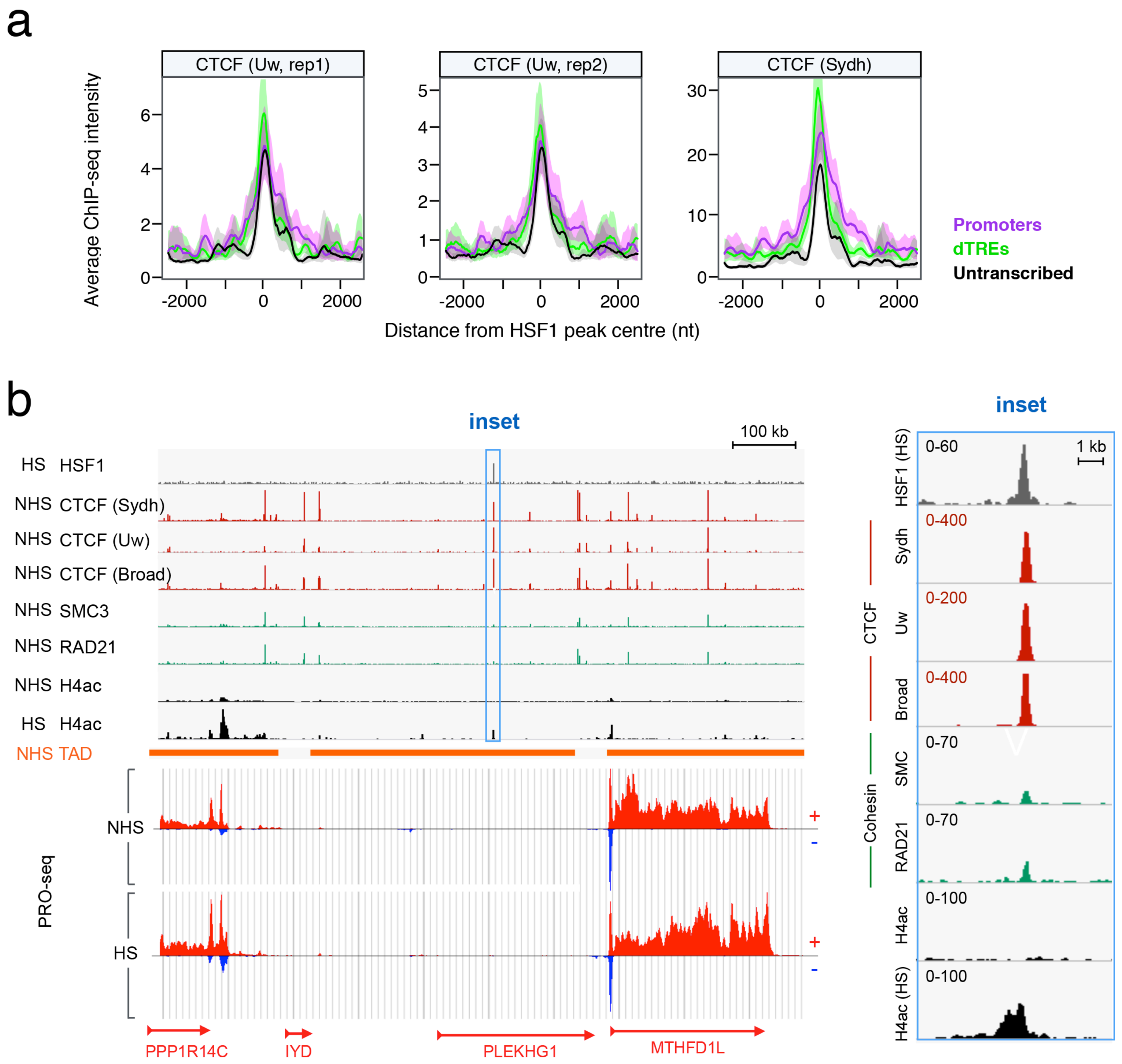

Supplementary Figure 8. HSF1 localizes to CTCF-enriched regions at untranscribed chromatin. a) Average CTCF ChIP-seq signal intensity at HSF1-targeted promoters, dTREs and untranscribed regions shown with three additional ENCODE datasets (the laboratory who produced the CTCF ChIP-seq data is indicated above each panel). **b)** Browser shot image of CTCF binding site in an untranscribed, topologically associated domain (TAD), and heat-induced histone H4 acetylation as well as binding of HSF1 at the CTCF-containing locus. The ChIP-seq datasets for CTCF, SMC3 and RAD21 were generated by the ENCODE[1], and the TADs obtained from the Hi-C data by Rao and co-workers[4].

Supplementary Methods

Identification of DNA elements and gene groups

Enriched DNA sequences at HSF1 target sites were queried with MEME-ChIP[5] from a 120-bp window centered on HSF1 ChIP-seq peak summit point. To identify molecular functions and biological processes associated with up- or down-regulated genes, we used gene ontology annotation by DAVID tool[6], selecting non-redundant genes in each group for further analyses.

Visualization of genome-wide data

The ChIP-seq and PRO-seq datasets were visualized using Integrative Genomics Viewer[7] and an in-house tool (Hoojong Kwak, Cornell University, Ithaca, USA). The scale of y-axis is equal when comparing signal intensities of any given factor between NHS and HS conditions. Unmappable genomic regions are shown in the bottom of the figure when appropriate and are derived from the RefGene databases.

Downloaded datasets

The presence, absence, and intensity of functional chromatin marks in non-stressed K562 cells were analyzed using datasets downloaded from ENCODE[1] (http://hgdownload-test.cse.ucsc.edu/goldenPath/hg19/encodeDCC/). GRO-cap[3] (GSE60456), TBP ChIP-nexus[2] (GSE55306) and Hi-C[4] (GSE63525), previously prepared from non-stressed K562 cells, were obtained from GEO.

Supplementary References

1. Consortium EP, ENCODE Project Consortium. A user's guide to the encyclopedia of DNA elements (ENCODE). *PLoS Biol.* **9**, e1001046 (2011).
2. He, Q., Johnston, J., Zeitlinger, J. ChIP-nexus enables improved detection of in vivo transcription factor binding footprints. *Nat. Biotechnol.* **33**, 395-401 (2015).
3. Core, L.J., Martins, A.L., Danko, C.G., Waters, C.T., Siepel, A., Lis, J.T. Analysis of nascent RNA identifies a unified architecture of initiation regions at mammalian promoters and enhancers. *Nat. Genet.* **46**, 1311-1320 (2014).
4. Rao, S.S., Huntley, M.H., Durand, N.C., Stamenova, E.K., Bochkov, I.D., Robinson, J.T., Sanborn, A.L., Machol, I., Omer, A.D., Lander, E.S., Aiden, E.L. A 3D map of the human genome at kilobase resolution reveals principles of chromatin looping. *Cell* **159**, 1665-1680 (2014). Erratum in: *Cell* **162**, 687-688 (2015).
5. Machanick, P., Bailey, T.L. MEME-ChIP: motif analysis of large DNA datasets. *Bioinformatics* **27**, 1696-1697 (2011).
6. Huang, da W., Sherman, B.T., Lempicki, R.A. Systematic and integrative analysis of large gene lists using DAVID bioinformatics resources. *Nat. Protoc.* **4**, 44-57 (2009).
7. Thorvaldsdóttir, H., Robinson, J.T., Mesirov, J.P. Integrative Genomics Viewer (IGV): high-performance genomics data visualization and exploration. *Brief Bioinform.* **14**, 178-192 (2013).
